# The effectiveness of virtual reality for people with mild cognitive impairment or dementia: a meta-analysis

**DOI:** 10.1186/s12888-019-2180-x

**Published:** 2019-07-12

**Authors:** Oksoo Kim, Yanghee Pang, Jung-Hee Kim

**Affiliations:** 1 College of Nursing, Ewha Womans University, 52 Ewhayeodae-gil, Seodaemun-gu, Seoul, 03760 South Korea; 2 Associate Professor, Department of Clinical Nursing, College of Nursing, The Catholic University of Korea, 222 Banpo-daero, Seocho-gu, Seoul, 06591 South Korea

**Keywords:** Mild cognitive impairment, Dementia, Meta-analysis, Virtual reality

## Abstract

**Background:**

Virtual Reality (VR) is increasingly used in health-related fields and interventions using VR have the potential to be powerful tools in patient management. The aim of this study was to synthesize the effects of VR interventions for people with mild cognitive impairment (MCI) or dementia.

**Methods:**

Electronic databases were searched to identify studies that used an experimental design to investigate VR intervention outcomes for patients with MCI or dementia. Studies were excluded if the intervention did not focus on VR, if relevant quantitative outcomes were not reported, or if the intended study purpose was assessment or diagnosis. Data were extracted and analyzed from studies that met criteria. To synthesize the intervention effect sizes (ES), we used random effects models to accommodate heterogeneity in the main effect and sub-group analyses. To identify the potential reason for heterogeneity and compare ES according to the moderator variables, subgroup analyses were conducted based on study characteristics and intervention outcomes.

**Results:**

Data from eleven studies that met eligibility criteria were analyzed. VR intervention delivered to participants with MCI or dementia produced small to medium effects (ES = 0.29, CI = 0.16, 0.42). The ES for studies using semi-immersive technology (ES = 0.37, CI = 0.25, 0.49) was greater than the studies using full-immersive VR (ES = 0.03, CI = -0.14, 0.21). The results showed small-to-medium effects for VR interventions affecting key outcome variables such as cognition (ES = 0.42, CI = 0.24, 0.60) and physical fitness (ES = 0.41, CI = 0.16, 0.65).

**Conclusion:**

VR interventions, particularly of the semi-immersive type, are useful for people with MCI or dementia. These results should contribute to the establishment of practical guidelines for VR interventions for patients with cognitive decline.

## Introduction

Dementia is one of the major causes of disability and dependency among older people. According to the World Health Organization, the number of patients with dementia worldwide is currently estimated at 47 million, and this is expected to increase to 75 million by 2030 and nearly triple by 2050 [1]. The estimated cost of dementia to the global economy increased by 35% from 2010 to 2015 [2]. Mild cognitive impairment (MCI) is an intermediate stage of cognitive change, between normal aging and dementia; the distinction between MCI and dementia is determined by the severity of cognitive decline leading to functional impairment [3]. Among elderly Chinese, the prevalence of overall MCI was 20.8%, with higher rates in rural than in urban areas [4]. In a representative Spanish sample, the overall prevalence of MCI was 9.6%, with higher rates in older people and women than in younger people and men [5].

People with cognitive decline report decreased stress when using virtual reality (VR) for both stimulation and relaxation [6]. Patients with cognitive decline may critically benefit from the implementation of VR technology interacting in a multisensory fashion through quasi-naturalistic, realistic stimuli [6–9]. VR interventions have increasingly been applied to address phobias, stress, and anxiety in psychotherapy [6] and behavioral therapy [10] as well as in the diagnosis of dementia [11]. VR offers cost-effective, accessible, flexible, and comprehensive interventions for patients who have difficulty attending outpatient appointments due to distance, lack of transport, or disability [6, 12]. Given the increasing use of VR in health-related fields, interventions using VR have the potential to be powerful tools in patient management. VR integrates real-time computer graphics, body tracking devices, visual displays, and other sensory inputs, which can be utilized to provide long-term and individualized care for patients with dementia [6]. To our knowledge, guidelines on VR intervention development and the length and dose of effective VR interventions are also lacking. Consequently, this study aimed to synthesize the effects of VR for patients with MCI or dementia.

A systematic review by Coyle et al. [13] examined computerized and VR cognitive training with individuals at high risk for cognitive decline. Further, the effectiveness of multisensory stimulation for individuals with dementia [14] and the use of VR to promote motor recovery in stroke rehabilitation [15] have been reviewed. Other meta-analyses have reviewed the efficacy of VR in examining behavioral assessments [10] and cognitive rehabilitation for brain injuries [16]. However, because the existing literature has mainly focused on the diagnosis of dementia using VR, studies examining the effects of VR intervention are limited and the literature in this area is in its infancy.

A meta-analysis allows researchers to review and assess knowledge in important areas and facilitates evidence-based practice. This statistical technique allows researchers to derive comprehensive results and an objective verification of intervention effectiveness by applying statistical methods to the results of existing scientific studies [17]. By using a meta-analysis, researchers can synthesize findings from studies that are methodologically different to compare the effects of a particular intervention. Important questions in a meta-analysis are whether the methodological, contextual, or substantive differences are related to variation in effect-size parameters.

Virtual environments are defined as “interactive, virtual image displays enhanced by special processing and by non-visual display modalities: to convince users that they are immersed in a synthetic space” [18]. VR is an artificial environment that is created with software and hardware and presented in which users can have close-to-reality experiences [7, 9, 19] VR resembles real-life situations and patients feel the sensation of “presence” or “being there” [7]. Giving immediate performance feedback and offering a personalized environment as well as a higher degree of similarity with the real world could further engage participants [8].

VR systems can be classified into several types according to the virtual environment (e.g., desktop, goggles-and-gloves, large screen, virtual room) and type of interaction technique (e.g., full- immersive, semi-immersive, non-immersive, and passive or active interaction) [6]. VR systems consist of 3D displays that effectively place the patient inside the virtual environment for the highest level of immersion [8]. The simplest form of VR is a 3-D image that can be explored interactively on a personal computer, usually by manipulating keys or the mouse, so that the content of the image moves in some direction or zooms in or out. However, more sophisticated approaches include wrap-around display screens, actual rooms augmented with wearable computers, and haptic devices that let people feel the display images [19].

During VR training, including Wii, participants use wireless controllers to interact with the on-screen avatars via the VR motion detection system. VR training using visual feedback enables participants to see their own movements, a feature that has helped patients adjust their misaligned body center caused by body image damage. In addition, studies show that training serves as a catalyst to active task participation by inducing interest and pleasure and providing immediate visual feedback on performance to enhance motor skills [20, 21].

The aim of this study was to synthesize the effects of virtual reality for people with MCI or dementia. The specific research questions considered are as follows:

- What is the overall magnitude of the effect of VR for patients with MCI or dementia?
- Which level of dementia stage (MCI, dementia) and intervention setting (community, institution) have the most influence on VR effect size?
- Which VR methodology type (game, task) and interaction type (semi-, full-immersive) have the most influence on intervention effect size?
- Which study method variables (random allocation) and evaluation methods (self-reported, observer) have the most influence on VR effect size?

## Methods

### Data sources and searches

A systematic search using the following designated keyword combinations were used to search eligible articles: (“dementia” OR “Alzheimer’s disease” OR “mild cognitive impairment” OR “cognitive impairment”) AND (“virtual reality” or “virtual”) without time limit. This study was conducted following the guidelines recommended by the Preferred Reporting Items for Systematic Reviews and Meta-Analyses statement [22]. To identify relevant studies, researchers conducted a systematic search of the following electronic databases: EBSCO, PubMed, ScienceDirect, and comprehensive Korean databases including the Korean Medical Database, Research Information Sharing Service, and National Digital Library.

The search results were restricted to studies meeting the following inclusion criteria: [1] included a VR intervention; [2] participants had MCI, dementia, or Alzheimer’s disease (AD); [3] utilized an experimental design including a control group, case series, randomized or non-randomized design; and [4] were available in full text in English or Korean. Studies were excluded if the intervention did not focus on VR, if relevant quantitative outcomes were not reported, or if the intended purpose was assessment or diagnosis. Further, conference proceedings, case reports, and literature reviews were excluded because they failed to yield effect sizes.

### Screening process

The screening process was conducted by a researcher (JH) and a research assistant (ES). To avoid selection bias, the initial online search was conducted independently by JH and ES. After exclusion of duplicate studies, titles and abstracts were reviewed; if an abstract was considered relevant or ambiguous, the full text was reviewed jointly, using inclusion and exclusion criteria. Any disagreements in study selection were resolved by discussion until consensus was reached.

**Fig. 1:**
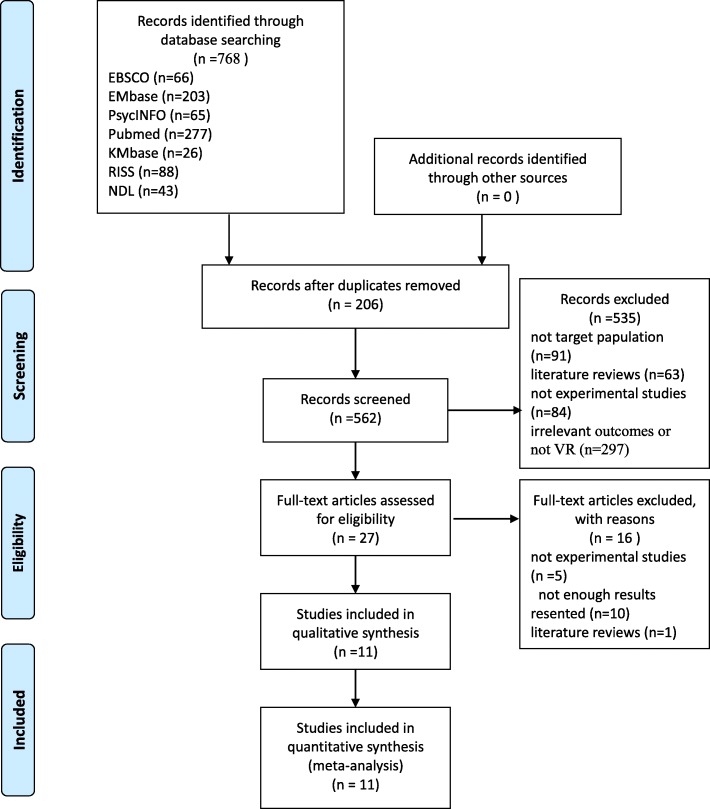
PRISMA Flow Chart

The initial electronic database search yielded 768 potentially relevant articles; however, 206 duplicates were excluded. Then, the remaining 562 titles and abstracts were scanned to identify potentially relevant studies. Five-hundred thirty-five did not match the inclusion criteria due to: different target population (*n* = 91), literature reviews (*n* = 63), non-experimental studies (*n* = 84), or irrelevant/non-VR outcomes (*n* = 297). Next, 27 full reports were obtained. Of these, 16 were excluded because they either employed non-experimental studies (*n* = 5) [23–27], presented insufficient results (*n* = 10) [28–36], or reviewed the literature [37]. As a result, data were extracted from a total of 11 studies that met inclusion and exclusion criteria (Fig. 1).

### Methodological quality assessment

We assessed the methodological quality of the included studies using the Risk of Bias Assessment Tool for Nonrandomized Studies (RoBANS), which comprises six domains: participant selection, confounding variables, measurement of exposure, blinding of outcome assessments, incomplete outcome data, and selective outcome reporting [38]. Two authors (OS and JH) assessed the RoBANS for all 11 studies and rated eligible studies as having either a high risk, low risk, or uncertain risk of bias. Any disagreements were resolved until consensus was reached. Eight studies were evaluated as high risk for a potential bias in the blinding of outcome assessments. Five studies had a high risk of having confounding variables. Six studies had a risk of exposure measurement bias.

### Data extraction

Data were extracted from studies that met our inclusion criteria by two reviewers (JH and OS) independently. A coding manual including information regarding effect size calculations and study characteristics was developed. The characteristics extracted from each study were: design (year of publication, country of origin, sample size, control group, and evaluation level), patient characteristics (age, dementia stage, and residential status), intervention characteristics (impairment feature, VR environment, VR type, type of interaction technique, session length, and dose), and intervention outcomes. The type of interaction was categorized into three levels of immersion. Interaction with a PC monitor, keyboard, and mouse were non-immersive [39, 40], more sophisticated graphics with larger surface displays were semi-immersive, and 3D displays were coded as full-immersive (i.e., highest level of immersion) [7, 40]. Intervention outcomes in each study were classified as physical fitness, cognition, emotion, execution, and feasibility. The evaluation of physical fitness included balance ability, gait, and fall measures. Cognition included cognitive ability, memory, concentration, orientation, recall, wording, and attention. Emotion included anxiety, psychological well-being, depression, and apathy measures. Verbal response and performance errors were classified in execution. Feasibility usually evaluated barriers to, and facilitators of intervention use, and users’ reported experiences with the intervention.

Any discrepancies that arose between the two reviewers were resolved by discussing the coding differences. As a result, no discrepancies remained between researchers for any items in this study, therefore, it was not necessary to calculate the inter-coder reliability.

### Data synthesis and statistical analyses

All effect sizes (ES) were calculated using Comprehensive Meta-Analysis version 2 (Biostat, Englewood, New Jersey). Fixed- and random-effects models address the problem of heterogeneity in distinct ways. A fixed-effects model assumes that primary studies have a common ES. A random-effects model estimates the distribution in the mean ES, assuming that each primary study has a distinct population. We used a random-effects model to accommodate heterogeneity for the main effect and sub-group analyses.

It has been proposed a conditional random effects model in which the choice between models depends on a homogeneity test with the Q-statistic [41]. Significant Q statistics (*p* < .01) were identified as heterogeneous. To identify the potential reason for heterogeneity and compare the ES according to the moderator variables, subgroup analyses were conducted based on study characteristics and intervention outcomes.

Evidence of publication bias was assessed by the “fail-safe N method,” which calculates how many missing studies are needed to nullify the effect [42, 43]. Because we needed a large number of studies to nullify the effect, publication bias was not a concern. In this study, the number of missing studies that would bring the *p*-value above .05 was 956 (Z value for alpha = 1.959).

To estimate ES of the studies, we used distinct data formats with appropriate formulae: one-group (pretest and posttest) and control group (pretest and posttest). Borenstein et al. [17] commented that systematic reviews could include studies that used independent and matched groups because the ES (d or g) has the same statistical meaning regardless of the study design. Therefore, there should be no technical barriers to including studies with different designs in the same analysis. Utilizing Cohen [44], effect sizes of 0.2, 0.5, and 0.8 were considered small, medium, and large, respectively.

## Results

### Study characteristics

Three of the 11 studies (27.2%) assessed MCI or questionable dementia, and seven (63.6%) focused on patients with dementia or AD. Only one study addressed both MCI and dementia. Patients’ age range was 63–89 years. Most studies (72.7%) were conducted in the community; two (18.2%) were conducted in an institute and one (9%) was conducted in both the community and an institute. Interventions were delivered using various VR platforms including a laptop, game controller, VR auditorium, motion sensor, Wii, virtual partner, virtual city, or 3D LCD glasses. Semi-immersive type (82.8%) was the most commonly used and a full-immersive type was applied in two studies (18.2%; Table 1).

**Table 1:** Characteristics of Studies included in the Analysis (*N* = 11)

Author(year)	Nation	Mean Age (Trt / Cont)	Sample size	Dementia	Institution	Impaired feature	VR environment	VR Methodology type	Interaction technique	Session length	Dose (Total number of training min)	Control group	Evaluation level
Burdea (2015) [45]	USA	63.4	10	dementia	community	cognition	laptop, game controller pendants	game	semi	20~40	1840	no control	self-reported
Flynn (2003) [46]	UK	69.6	6	dementia	mixed	behavior	VR auditorium	task	semi	20	50	no control	self
Moyle (2017) [47]	Australia	89	10	dementia	Institute	mood	motion sensor	task?	full		35	no control	observer
Man (2011) [48]	Hong Kong	80.3/ 80.2	20/14	CDR = 0.5 questionable dementia	community	cognition	joy stick or keyboard	task	semi	30	300	psycho education	self reported
Lee (2017) [24]	Korea	71.8/ 69.7	15/15	AD	community	Physical fitness	Wii, Motion tracking	task	semi	40	1440	cognitive therapy	observer
Park (2016) [49]	Korea	71.3/ 72.2	10/10	dementia	institute	Physical fitness	Wii, Motion tracking	task	semi	15	360	cognitive therapy	self reported
Hwang (2017) [50]	Korea	74.1/ 70.1	24/24	MCI	community	cognition, balance	Motion tracking	task	semi	30	600	occupation therapy	observer
Schwenk (2016) [51]	USA	77.8/ 77.9	11/9	MCI	community	gait, balance	Abartar, sensor	task	semi	45	360	no training	self and observer
Lancioni (2015) [52]	Italy	83.6/ 82	6/ 11	AD	community	attention	microswitch, virtual partner	microswich activation	semi	5	485	photo, video	observer
Serino (2017) [53]	Italy	86.6/ 88.7	10/10	AD	community	cognition	gamepad, virtual city	task	semi	20	200	cognitive therapy	observer
Manera (2016) [8]	France	75/ 75	28/28	dementia, MCI	community	attention	3D LCD shutter glasses	task	full	20	20	paper condition	self and observer

*Abbreviations*: *AD* Alzheimer Disease, *MCI* Mild Cognitive Impairment, *VR* Virtual Reality, *Trt* Treatment, *Cont* Control, *CDR* Clinical Dementia Rating

### Overall analysis

**Fig. 2:**
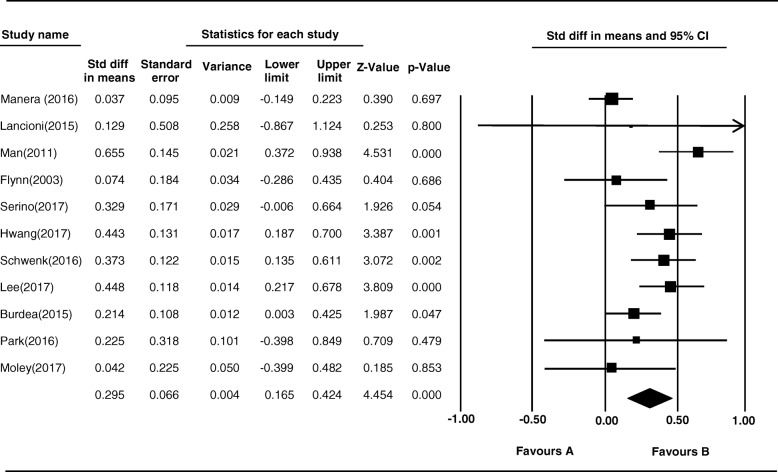
Forest Plots for Primary Studies

When the studies were combined in the meta-analysis, high heterogeneity was observed (Q = 21.572, *p* < .001). Concerning MCI or AD, VR produced small-to-medium effect sizes using the random-effects model (ES = 0.29, CI = 0.16, 0.42) (Table 2 and Fig. 2).

**Table 2:** Overall Result of the Meta-Analysis using a Random Effects Model

N	−95%CI	ES	+ 95%CI	SE
11	0.16	0.29	0.42	0.06

*Abbreviations*: *CI* confidence interval, *ES* effect size, *N* number of studies, *SE* standard error

### Effect sizes per study characteristics

The meta-analysis provided 70 effect sizes from 11 primary studies. The results of a meta-analysis of the moderators of effects in VR for patients with dementia are shown in Table 3. VR interventions for patients with MCI resulted in greater effects (ES = 0.40, CI = 0.23, 0.58) than did interventions for patients with dementia (ES = 0.35, CI = 0.18, 0.51), or for the mixed group (ES = 0.03, CI = -0.18, 0.25). Studies conducted in the community (ES = 0.33, CI = 0.21, 0.45) had larger effect sizes than those conducted in an institution (ES = 0.10, CI = -0.25, 0.46), or in both the community and an institution (ES = 0.07, CI = -0.28, 0.43). Six studies used two-group posttest, one study used a control group, and four studies used the one-group design. Regarding experimental and control group allocation, random allocation (ES = 0.36, CI = 0.18, 0.53) and no randomization (ES = 0.4, CI = 0.19, 0.61) showed small to moderate effects, which were larger than those with a one-group design (ES = 0.15, CI = -0.01, 0.33).

**Table 3:** Effect Sizes by Subgroup according to Study Characteristics

Subgroup	Categories	*k*		95%CI ES	+ 95%CI		p	SE
Dementia stage	Dementia	44		0.18	0.35	0.51	.000	0.07
	MCI	18		0.23	0.40	0.58	.000	0.08
	Dementia+ MCI	8		−0.18	0.03	0.25	.749	0.10
Institution	Community	61		0.21	0.33	0.45	.000	0.06
	Mixed	5		−0.28	0.07	0.43	.686	0.14
	Institution	4		−0.25	0.10	0.46	.575	0.18
Methodology type	Game	9		0.01	0.21	0.42	.047	0.10
	Task	63		0.20	0.32	0.45	.000	0.06
Type of interaction technique	Semi	60		0.25	0.37	0.49	.000	0.05
	Full	10		−0.14	0.03	0.21	.684	0.09
Randomization	Yes	41		0.18	0.36	0.53	.000	0.08
	No	13		0.19	0.40	0.61	.000	0.44
	NA	16		−0.01	0.15	0.33	.065	0.08
Evaluation	Self-reported	29		0.10	0.31	0.52	.003	0.10
	Observer	38		0.19	0.30	0.42	.000	0.05

*Abbreviations*: *CI* confidence interval, *ES* effect size, *k*, number of effect size, *SE* standard error

For methodology type, VR using a task (ES = 0.32, CI = 0.20, 0.45) resulted in a larger effect than VR using a game (ES = 0.21, CI = 0.01, 0.42). Regarding type of interaction technique, studies using semi-immersive technology (ES = 0.37, CI = 0.25, 0.49) had a greater effect than studies using full-immersive technology (ES = 0.03, CI = -0.14, 0.21). Lastly, regarding type of evaluation, effect sizes were higher for self-reported evaluation (ES = 0.31, CI = 0.10, 0.52) than for observer-reported evaluation (ES = 0.30, CI = 0.19, 0.42).

### Effect sizes according to intervention outcomes

Regarding the VR intervention outcome, the effect size of cognition (ES = 0.42, CI = 0.24, 0.60) was higher than that of physical fitness (ES = 0.41, CI = 0.16, 0.65), emotion (ES = 0.14, CI = -0.07, 0.36), execution (ES = 0.07, CI = -0.34, 0.49) or feasibility (ES = 0.12, CI = -0.10, 0.34) (Table 4).

**Table 4:** Effect Sizes by Subgroup according to Intervention Outcomes

Subgroup	Categories	*k*	−95%CI	ES	+ 95%CI	p	SE
Intervention Outcomes	Physical fitness	12	0.16	0.41	0.65	.001	0.12
	Cognition	36	0.24	0.42	0.60	.000	0.06
	Emotion	10	−0.07	0.14	0.36	.198	0.11
	Execution	6	−0.34	0.07	0.49	.335	0.21
	Feasibility	7	−0.10	0.12	0.34	.302	0.10

*Abbreviations*: *CI*, confidence interval; *ES*, effect size; *k*, number of effect size; *SE*, standard error

## Discussion

The aim of this study was to synthesize the effects of 11 experimental studies that used VR interventions with patients with MCI or dementia. The results showed small-to-medium positive effects for VR interventions on key outcome variables such as physical fitness, cognition, and emotion. This indicates that VR interventions can positively affect various clinical outcomes in patients with cognitive impairment, implying that VR interventions improve cognitive and routine functions by stimulating patients’ brains [6, 54].

In this study, heterogeneity was identified among the individual studies, so a random-effects model was applied. Subgroup analysis was performed per the characteristics of the individual studies. This meta-analysis used a common metric to determine the magnitude of effects in three different data formats using two-group posttest (standardized mean difference), control group (pretest and posttest), and the one-group (pretest and posttest). All intervention effects were calculated and a pooled estimate of the standard deviation for the population was used. As the ES for random allocation (0.36) and no randomization (0.40) among the study with control group were medium, VR interventions were considered useful for people with MCI or dementia.

The VR intervention effects were greater in those examining patients with MCI as compared to those examining patients with dementia or both dementia and MCI. In addition, the outcome effects were greater when the intervention was conducted in the community as compared to in an institution or both the community and an institution. Consequently, we posit that VR interventions are most effective with patients with MCI in a community setting. Concerning methodology, a task was more effective than a game. Moreover, semi-immersive technology was more effective than full-immersive technology including 3D display and HMD. Perhaps VR-based intervention programs provide more diverse, comprehensive, and secure functions but can be complex and difficult for older adults to use [55].

Recently, commercialized VR has developed rapidly and is readily available [6]. However, VR interventions for older adults with reduced vision or other sensory problems may be unsafe [56]. Therefore, it is necessary to consider a methodology type and an interaction technique that will result in the safe implementation of a VR program for people with sensory and cognitive impairment, including older patients with dementia.

The VR intervention effect in the cognition category was small to medium, which is consistent with the results of a systematic review by Coyle et al. [13]. Coyle and colleagues showed that a VR intervention moderately improved the cognitive functioning of participants with cognitive impairment. Further, prior studies showed VR interventions were effective in improving the physical functions and walking speed of community-dwelling patients who had a stroke [54, 57]. This suggests that a VR physical fitness intervention may effectively improve the functioning of patients with MCI or dementia.

The intervention effect was slightly higher when the evaluation method was self-reported as compared to observer-reported. For people with cognitive impairment, it is essential to confirm the effectiveness of the program using both subjective evaluations from self- and other-reports [58]. More objective evaluation methods through observation should be implemented in future studies.

VR could also be adapted to patients’ needs and characteristics in performing activities, tasks, and tests [7]. VR is an effective intervention for patients with AD when it can be performed safely [6, 12]. Patients with MCI or dementia reported a VR task to be more satisfying, secure, comfortable, and less anxiety provoking than a paper-based task [8]. Older patients with dementia experience impaired sensory stimulation due to impaired cognitive function and aging, which can result in anxiety, agitated behaviors, and impaired social functioning [14]. In a VR condition, patients experience various forms of sensory stimulation in a comfortable, safe, immersion environment that can promote functional learning as well as the transfer of learned functions [14].

VR interventions positively affected the cognitive and physical functioning of patients with MCI or dementia. However, because most existing studies have focused on the diagnosis of dementia using VR, studies examining the effects of VR intervention are limited. There is also a lack of guidelines on the VR development process and the length and dose of effective interventions. Current study results should contribute to the establishment of practical guidelines for VR intervention for patients with cognitive decline. Because VR is cost-effective, flexible, comprehensive, and useful for patient-centered care, it is likely that the scope of VR activities will grow with further technological innovation [13].

## Conclusion

This meta-analysis integrated individual studies about VR interventions for patients with cognitive impairment because VR is cost-effective, flexible, comprehensive, and potentially useful for patient-centered care. This study showed small-to-medium effects on key outcome variables such as physical fitness, cognition, and emotion. Interventions using VR could be useful for people with MCI or dementia. These results should contribute to the establishment of practical guidelines for VR intervention for patients with cognitive decline. This study had some limitations. First, most analyzed studies did not produce a significant ES because the sample sizes were small and pilot tests were included. Second, the methodological quality of some studies was low (e.g., not randomizing or blinding assessment outcomes). Despite these limitations, the results presented in meta-analysis suggest a basis for evidence-based interventions for patients with MCI or dementia. Findings also underscore the need for comprehensive guidelines to develop and implement safe, effective VR interventions to improve functional outcomes for people with cognitive impairment.

## Data Availability

The dataset used and analyzed during the current study is available from the corresponding author on reasonable request.

## Abbreviations

AD: Alzheimer Disease
CDR: Clinical Dementia Rating
CI: Confidence interval
Cont: Control
ES: Effect size
MCI: Mild Cognitive Impairment
MCI: Mild cognitive impairment
N: Number of studies
RoBANS: Risk of Bias Assessment Tool for Nonrandomized Studies
SE: Standard error
Trt: Treatment
VR: Virtual Reality
VR: Virtual reality
